# Connexin Expression in Pituitary Adenomas and the Effects of Overexpression of Connexin 43 in Pituitary Tumor Cell Lines

**DOI:** 10.3390/genes13040674

**Published:** 2022-04-12

**Authors:** Bruno Nunes, Helena Pópulo, José Manuel Lopes, Marta Reis, Gilvan Nascimento, Ana Giselia Nascimento, Janaína Fernandes, Manuel Faria, Denise Pires de Carvalho, Paula Soares, Leandro Miranda-Alves

**Affiliations:** 1 Laboratory of Experimental Endocrinology—LEEx, Institute of Biomedical Science, Federal University of Rio de Janeiro, Rio de Janeiro 21941-902, Brazil; nunesb.bioufma@hotmail.com (B.N.); dencarv@gmail.com (D.P.d.C.); leandro.alves@icb.ufrj.br (L.M.-A.); 2 Postgraduate Program in Endocrinology, Faculty of Medicine, Federal University of Rio de Janeiro, Rio de Janeiro 21941-902, Brazil; 3 Laboratory of Endocrine Physiology, Doris Rosenthal, Carlos Chagas Filho Institute of Biophysics, Federal University of Rio de Janeiro, Rio de Janeiro 21941-902, Brazil; 4 Institute for Research and Innovation in Health, University of Porto, 4200-135 Porto, Portugal; hpopulo@ipatimup.pt (H.P.); jmlopes@ipatimup.pt (J.M.L.); ines.marta@gmail.com (M.R.); 5 Institute of Molecular Pathology and Immunology of the University of Porto (IPATIMUP)—Cancer Signalling & Metabolism, 4200-135 Porto, Portugal; 6 Department of Pathology, Medical Faculty of the University of Porto, 4200-319 Porto, Portugal; 7 Centre of Clinical Research (CEPEC), President Dutra Hospital of Federal University of Maranhão (UFMA), São Luís 65020-600, Brazil; gilvancortes@uol.com.br (G.N.); mfaria1949@gmail.com (M.F.); 8 Endocrinology Service, President Dutra Hospital of Federal University of Maranhão (UFMA), São Luís 65060-600, Brazil; 9 Pathology Service, President Dutra Hospital of Federal University of Maranhão (UFMA), São Luís 65020-070, Brazil; anagiselia@gmail.com; 10 NUPEX, Polo Duque de Caxias, Universidade Federal do Rio de Janeiro, Rio de Janeiro 25240-005, Brazil; janainaf@biof.ufrj.br; 11 Postgraduate Program in Pharmacology and Medicinal Chemistry, Institute of Biomedical Science, Federal University of Rio de Janeiro, Rio de Janeiro 21941-902, Brazil

**Keywords:** connexins, pituitary neuroendocrine tumors, apoptosis

## Abstract

Gap junction intercellular communication (GJIC) is considered a key mechanism in the regulation of tissue homeostasis. GJIC structures are organized in two transmembrane channels, with each channel formed by connexins (Cxs). GJIC and Cxs expression alterations are related to the process of tumorigenesis in different cell types. Pituitary neuroendocrine tumors (PitNETs) represent 15–20% of intracranial neoplasms, and usually display benign behavior. Nevertheless, some may have aggressive behavior, invading adjacent tissues, and featuring a high proliferation rate. We aimed to assess the expression and relevance of GJIC and Cxs proteins in PitNETs. We evaluated the mRNA expression levels of Cx26, 32, and 43, and the protein expression of Cx43 in a series of PitNETs. In addition, we overexpressed Cx43 in pituitary tumor cell lines. At the mRNA level, we observed variable expression of all the connexins in the tumor samples. Cx43 protein expression was absent in most of the pituitary tumor samples that were studied. Moreover, in vitro studies revealed that the overexpression of Cx43 decreases cell growth and induces apoptosis in pituitary tumor cell lines. Our results indicate that the downregulation of Cx43 protein might be involved in the tumorigenesis of most pituitary adenomas and have a potential therapeutic value for pituitary tumor therapy.

## 1. Introduction

The cell-mediated communication by gap junctions-gap junctions intercellular communication (GJIC) is considered a key mechanism in tissue homeostasis and regulation of cell growth and differentiation [1,2]. Gap junctions are formed by intercellular channels that connect the cytoplasm of neighboring cells, through which is possible the direct exchange of molecules that are essential for cell function with a relative molecular mass of up to 1.2 kDa [3,4].

Gap junctions are present in almost all tissue cells both during embryonic development and adulthood [2,5]. Almost all cells are interconnected via gap junction channels, except for platelets, erythrocytes, fully differentiated skeletal muscle cells, and spermatozoa [5,6]. Gap junctions are involved in many cellular processes, such as metabolic support, electrical coupling, control of gene expression, cell proliferation, differentiation, survival, and apoptosis [7,8]. In the anterior pituitary, there are two main cell types, the endocrine cells that are classified by the hormones that they produce, and nonendocrine cells, which include stem/progenitor and folliculostellate cells (FS cells) [9,10]. Intercellular communication via gap junctions, has been described both between endocrine-endocrine and endocrine-nonendocrine cells [9,10].

Gap junctions are formed by two transmembrane hemichannels (connexons) that are present in each adjacent cell [2,3]. The hemichannels are formed by the oligomerization of six proteins, known as connexins (Cxs) [2,8]. Over 21 different isoforms of Cxs have been characterized in humans, with Cx43 as the most ubiquitously expressed and widely studied connexin in mammals [2,3]. Cxs are closely linked to healthy development and homeostasis and can also influence tissue homeostasis by interfering with channel-forming capabilities, including the signaling which may occur at the plasma membrane, in the cytoplasm, or even in the nucleus [11,12]. The aberrant expression of connexin and gap junctions is associated often with a tumorigenic phenotype [13].

Gap junction communication and connexin expression have been reported to be associated with tumorigenesis in different cell types, possessing both pro- and anti-tumorigenic functions [5,14,15,16]. Decreased expression and/or functions of Cxs have been described in many cancer cell lines and solid tumors, including liver, prostate, breast cancer, and melanoma [17,18].

Studies in mice and humans indicated that in many early-stage tumors, connexins are lost or mislocalized and their re-expression has been shown to reduce cell proliferation and partial reversion of epithelial to mesenchymal transition events in many different cancer cell lines, inhibiting tumor growth [11,13,14,19,20]. Moreover, an increasing number of studies support the therapeutic potential of targeting Cx43 as an option for tumor treatment [11,13,20,21,22].

In the pituitary, the intercellular communication mechanism remains poorly characterized [10,23]. The role of gap junctions and connexins expression in the pituitary neuroendocrine adenomas tumorigenesis process has been scarcely addressed. Pituitary neuroendocrine tumors (PitNETs) represent up to 15–20% of intracranial neoplasms, with a prevalence of 0.1% in the overall population. PitNETs are usually benign and can associate with endocrine and nonendocrine signs [24,25]. Although usually benign, some show aggressive clinical behavior; these tumors may cause significant morbidity because of their expanding size, hemorrhage, invasion to surrounding structures, and/or inappropriate pituitary hormone secretion [25,26].

PitNETs are classified into clinically functioning and non-functioning (NF). Functional tumors cause clinical syndromes that are associated with the hypersecretion of a hormone. According to the main hormone that is produced, PitNETs are classified in somatotroph adenomas that hyper secrete growth hormone (GH), leading to features of acromegaly; lactotroph adenomas that result in hyperprolactinemia; corticotroph adenomas that hyper secrete adrenocorticotropic hormone (ACTH), leading to features of hypercortisolism, such as Cushing’s disease; thyrotroph adenomas that hyper secrete thyroid-stimulating hormone (TSH); gonadotroph adenomas with hypersecretion of follicule-stimulating hormone/luteinizing hormone (FSH/LH) or with normal plasma levels. Non-functioning tumors do not cause signs and symptoms of hypersecretion, except hyperprolactinemia due to the hypothalamic disconnection [27,28].

PitNET can be further classified according to the tumor size in microadenomas (tumors with less than 10 mm), macroadenomas (tumors equal or greater than 10 mm and less than 40 mm), or giant adenomas (tumors equal to or greater than 40 mm) [25].

In addition, according to the invasive capacity of the surrounding structures and based on radiological, surgical, and histopathological criteria, tumors can be classified into invasive or non-invasive, [27,28,29]. PitNETs may invade one or both cavernous sinuses, and/or the bone and the respiratory mucosae breaching into the sphenoid sinus. Large tumors are often invasive. Although tumor size and invasion are related, only invasion is considered predictive of progression/recurrence [28].

The present study aimed to evaluate the levels of connexins mRNA and protein expression in a series of pituitary neuroendocrine adenomas, and the possible association with tumor clinic-pathological features. We also evaluated the effect of overexpression of Cx43 in pituitary tumor cell lines.

## 2. Materials and Methods

### 2.1. Clinical-Pathological Features

In the present study, 78 post-surgical pituitary neuroendocrine adenomas (PitNETs) were analyzed. The patients were followed by the Endocrinology Service and underwent transsphenoidal surgery at the Hospital of the Federal University of Maranhão, Brazil. The series included 38 NF-PitNETs, 27 somatotropinomas, and 13 corticotropinomas that were diagnosed using clinic-laboratory criteria and through imaging exams (magnetic resonance imaging) (Table 1). Tumor size was defined by radiological criteria as microadenoma (<10 mm), macroadenomas (>10 mm), or giant adenomas (>40 mm). Regarding invasiveness, PitNETs were classified as invasive or noninvasive according to Knosp’s classification [29]. The procedures followed the institutional ethical standards and were performed according to the Declaration of Helsinki. All the patients provided written informed consent. Normal human anterior pituitary samples were obtained at the autopsy of four patients of the Death Verification Service of Federal University of Maranhão, Brazil. These samples were examined using hematoxylin-eosin stain to exclude the possibility of incidental tumors. The research ethics committee of the University Hospital of the Federal University of Maranhão Brazil approved this study under the number 2.908.868.

### 2.2. RNA Extraction

Total RNA was extracted from the 60 available frozen PitNETs specimen samples and four normal pituitary specimens using the RNeasy Mini Kit (Qiagen, Redwood City, CA, USA) and treated with the DNase I kit (Biolabs, Ipswich, MA, USA) to remove possible contamination with genomic DNA, according to the manufacturer’s instructions. The RNA quantity was assessed using a Nanodrop 1000 spectrophotometer (Thermo Fisher Scientific, Waltham, MA, USA).

### 2.3. Quantitative Reverse Transcriptase-Polymerase Chain Reaction

The synthesis of cDNA was performed from 1 µg of total RNA from each sample using the High Capacity RNA-cDNA kit (Applied Biosystems, Waltham, MA, USA) in a final volume of 20 µL, following the manufacturer’s instructions. The reaction was performed using a Veriti 96-well thermal cycler. mRNA expression of Cx26, 32, and 43 were evaluated in the PitNETs adenomas and a pool of four normal human anterior pituitary samples. Quantitative reverse transcriptase-polymerase chain reaction (qRT-PCR) was performed using the Power SYBR Green PCR Master mix (Applied Biosystems, USA) and each sample was run in triplicate. The thermal cycling conditions consisted of one cycle at 50 °C for 2 min, and 95 °C for 10 min, followed by 40 cycles at 95 °C for 15 s, and 60 °C for 1 min. All the samples were normalized by a housekeeping gene, β-actin, and the amplification was performed using the 7500 Real-time PCR system (Applied Biosystems, USA). The relative gene expression was quantified using the 2^−ΔΔCt^ method and presented as the expression relative to that of the normal human anterior pituitary.

### 2.4. Immunohistochemistry Analysis

Sections of 4 µm thickness were obtained from 65 PitNETs tissue samples and 4 normal pituitary tissue samples that were fixed in 10% formalin and embedded in paraffin. The deparaffinized and rehydrated sections were then treated with 3% hydrogen peroxide to block endogenous peroxidase and with citrate buffer, pH 6.0, for five minutes in the microwave, for antigen retrieval. The samples were incubated overnight at 4 °C with the anti-connexin 43 primary polyclonal antibody (ab11370, Abcam; 1:1000), followed by incubation with the secondary antibody (biotinylated goat anti-polyvalent, Thermo Scientific, Waltham, MA, USA) for 1 h at room temperature. Then, the slides were treated with the chromogenic substrate DAB solution (3, 3′-diaminobenzidine, Dako, Santa Clara, CA, USA) and counterstained with Harris hematoxylin. The sections were mounted using Entellan (Millipore, Darmstadt, Germany) medium. A positive control, mouse heart tissue, and negative control, consisting of omission of the primary antibody, were included simultaneously in the experiments to ensure specificity and reliability of the immunoreactions. Immunohistochemistry results were semi-quantitatively evaluated by two observers. Immunoreactivity was evaluated separately concerning the intensity and extent of the staining. The intensity was equal to 0 when no staining was present, 1+ for weak, 2+ for moderate, and 3+ for strong staining. The extent was equal to 0 = 0–5%, 1 = 6–25%, 2 = 26–50%, 3 = 51–75%, and 4 = 76–100% of tumor cells that were immunostained. An immunoreactivity score was calculated by multiplying the intensity and extension scores. Thus, the scores were classified as negative (0), low (score ≤ 3), or moderate/high (score value 4–12).

### 2.5. Cell Culture

The mouse corticotrope tumor cell line AtT-20, secreting adrenocorticotropic hormone [30,31], was kindly provided by Dr. Ulrich Renner and Prof. Dr. Günter K. Stalla from the Clinical Neuroendocrinology Group, Max Planck Institute of Psychiatry, Germany. The rat Wistar-Furth pituitary tumor cell line GH3, secreting growth hormone and prolactin [32], was obtained from the Cell Bank of Federal University of Rio de Janeiro, Rio de Janeiro, Brazil. The cell lines were maintained in Dulbecco’s modified culture medium that was supplemented with 10% fetal bovine serum, antibiotics (100 U/mL penicillin and 100 µg/mL streptomycin, LGC Biotechnology, São Paulo, Brazil), and 5 mg/mL fungizone (amphotericin B) (Sigma-Aldrich, St. Louis, MO, USA) at 37 °C and 5% CO_2_.

### 2.6. Cx43 Transient Transfection

The GH3 cells were transfected using Lipofectamine 2000 (Life Technologies, Carlsbad, CA, USA), according to the manufacturer’s instructions, with an empty vector MClover2-N1 (EV) or mClover2-Cx43-7 (Cx43) containing the *Rattus norvegicus* connexin 43 coding sequence, both of which were a gift from Michael Davidson (Addgene plasmid #54538; #56532, respectively). The AtT-20 cells were transfected with MClover2-N1 (EV) and mClover2-Cx43-7 that were modified by directed mutagenesis using the QuickChange Lightning Site-Directed Mutagenesis Kit (Agilent Technologies, Santa Clara, CA, USA) to generate the mutation 1022A > G, thus creating a sequence that was identical to the wild-type sequence of Cx43 found in *M. Musculus*. The plasmid generated was then digested with the BamHI and EcoRI enzymes (Fermentas, Waltham, MA, USA). Plasmids were fused with a green fluorescent protein coding sequence (GFP). The cells were plated in 6-well plates at a density of 2.5 × 10^4^ (AtT-20) or 3 × 10^5^ (GH3) cells/well and incubated at 37 °C for 48 h. The DNA-Lipofectamine mixture was incubated for 20 min at room temperature and added to the cell cultures. The cells were then transfected with 1µg of vector and incubated for 24–48 h at 37 °C in the presence of 5% CO_2_.

### 2.7. Cell Count Assay

The transfected cells (EV and Cx43) were plated in 24-well plates at a density of 3 × 10^4^ (AtT-20) and 4 × 10^4^ (GH3) cells/well and incubated at 37 °C for 48 h. The transfected cells were washed three times with PBS 0.01 M, pH 7.4, and a solution of trypsin/EDTA 0.125% (Sigma-Aldrich, St. Louis, MO, USA) was added to the cells. The cell suspension was centrifuged for 5 min at 1800× *g*, and the cells were suspended in supplemented DMEM. Then, the culture medium was collected and diluted at 1:200, and the absolute cell count was performed using a Z2 Coulter particle counter (Beckman Coulter, Brea, CA, USA). Each experimental condition was evaluated in duplicate and repeated three times.

### 2.8. Cell Immunofluorescence

After transfection, the cells (EV and Cx43) were grown on coverslips for 48 h. The cells were washed two times in PBS 0.01 M, pH 7.4, and fixed in 4% paraformaldehyde for 20 min at room temperature. After gently washing with PBS, the cells were incubated in ammonium chloride (50 nM) for 10 min, followed by permeabilization with 0.2% Triton X-100 for 10 min and blocked with 5% bovine serum albumin for 30 min. The cells were then incubated overnight at 4 °C with the anti-connexin 43 polyclonal antibody (ab11370, Abcam; 1:200). Then, the cells were washed three times in PBS and incubated with Alexa-546-conjugated goat anti-rabbit secondary antibody (1:500, Molecular Probes, Carlsbad, CA, USA). Finally, the cells were incubated with DAPI (Sigma-Aldrich) for 5 min and mounted using VectaShield Mounting Medium (H-1000, Vector). Images were acquired using Axio Imager Microscopy-Zeiss.

### 2.9. Cell Cycle

The transfected cells (EV and Cx43) were plated in 24-well plates at a density of 3 × 10^4^ (AtT-20) and 4 × 10^4^ (GH3) cells/well and incubated at 37 °C for 48 h. The cells were quickly washed with calcium/magnesium-free balanced saline solution (BSS), detached with the aid of trypsin/EDTA 0.125% (Sigma, St Louis, MO, USA) at room temperature, and then centrifuged (5 min at 1800× *g*). The cells were washed with PBS and the pellet was resuspended in a hypotonic fluorescent solution (50 μg/mL propidium iodide (PI) and 0.1% Triton X-100 in 0.1% sodium citrate buffer). After 1 h incubation in the dark at 4 °C, the DNA content was measured by flow cytometry (FL-2) (FACSCalibur, Becton Dickinson, Franklin Lakes, NJ, USA). The relative proportions of cells with DNA content that was indicative of phase subG0/G1 (<2n), G0/G1 (2n), S (2n > 4n), and G2/M (4n) were determined. Data acquisition and analysis were done by CellQuest software version 3.1f. and sub diploid populations (<2n) were considered apoptotic. The results represent the average ± SEM of three independent experiments that were performed in triplicate.

### 2.10. TUNEL Assay

The cells were plated in 6-well plates at a density of 2.5 × 10^4^ (AtT-20) or 3 × 10^5^ (GH3) cells/well and incubated at 37 °C for 48 h. The cells were transfected with 1 µg of vector (EV and Cx43) and incubated for 24–48 h at 37 °C in the presence of 5% of CO_2_. Then, the floating and attached cells were collected and prepared for the cytospin technique. The cells were then fixed with 4% paraformaldehyde for 20 min at room temperature and then washed in PBS 0.01 M, pH 7.4. The cells were incubated in ammonium chloride (50 nM) for 10 min, followed by permeabilization with 0.1% Triton X-100 in 0.1% sodium citrate on ice for 2 min. A TUNEL reaction was performed using the ApopTag^®^ Red In Situ Apoptosis Detection Kit (Millipore, Darmstadt, Germany). The proportion of TUNEL-positive (apoptotic) nuclei was determined by counting at least 500 cells. Each experimental condition was evaluated in duplicate and repeated three times.

### 2.11. Western Blot Analysis

Total protein was extracted from GH3 (EV and Cx43) and AtT-20 (EV and Cx43) cells and quantified using a Pierce BCA protein assay kit (Thermo Scientific, Waltham, MA, USA). The cells were lysed for 15 min at 4 °C using RIPA buffer (1% NP-40 in 150 mM NaCl, 50 mM Tris pH 7.5, 2 mM EDTA) containing phosphatase and protease inhibitors. Equal amounts of protein (40 µg) were separated in 10% SDS-PAGE gels, followed by electrotransfer to nitrocellulose membranes (Millipore, Darmstadt, Germany). The membranes were blocked with 5% nonfat milk and incubated overnight at 4 °C with the anti-connexin 43 polyclonal antibody (ab11370, Abcam; 1:2000). The anti-α/β-tubulin (2148S, Cell Signaling, Danvers, MA, USA, 1:1000) was used as the loading protein control. Primary antibody binding was detected using an HRP-conjugated secondary antibody (anti-rabbit or anti-mouse, Santa Cruz Biotechnology, 1: 2000) and visualized by using a chemiluminescence substrate system (Millipore, Darmstadt, Germany). Densitometry analysis of the bands was performed using the Image J software (http://rsbweb.nih.gov/ij/docs/faqs.html accessed on 19 April 2020). The data were obtained from three independent experiments.

### 2.12. Statistical Analysis

Statistical analysis was performed using GraphPad PRISM 5.0 (GraphPad Software, Inc., La Jolla, CA, USA). To determine the significance of associations among the different variables, data were analyzed by Kruskal–Wallis test, Mann–Whitney U-test, χ^2^-test, Spearman’s correlation coefficient, and Pearson’s correlation coefficient. A *p*-value < 0.05 was considered statistically significant.

## 3. Results

### 3.1. mRNA Expression of Connexin 26, 32, and 43 in Pituitary Neuroendocrine Adenomas

The mRNA expression levels of Cx26, 32, and 43 in normal human pituitary and PitNETs adenomas samples were analyzed. The mRNA expression was successfully evaluated by qRT-PCR in 60 samples: 31 NF-PitNETs, 20 somatotropinomas, and 9 corticotropinomas. Variable levels of mRNA expression of Cx26, 32, and 43 were observed, as well as the expression of different connexins in the same tumor sample. PitNETs mRNA expression levels of Cx26, 32, and 43 are presented relative to the mRNA expression levels of a pool of four samples of the normal human pituitary.

The expression of Cx26 was found in 43 of the 60 (72%) pituitary adenomas samples: 20 of the 31 (65%) NF-PitNETs, 16 of the 20 (80%) somatotropinomas, and 7 of the 9 (78%) corticotropinomas. Cx26 expression was lower in NF-PitNETs and corticotropinomas compared with to the pool of normal pituitary. In somatotropinomas the median Cx24 expression was similar to the pool of normal pituitary (Table 2).

Cx32 was expressed in 43 of the 60 (72%) pituitary adenoma samples: 22 of the 31 (71%) NF-PitNETs, 16 of the 20 (80%) somatotropinomas, and 5 of the 9 (56%) corticotropinomas. All the PitNETs showed downregulation of Cx32 expression in comparison to the pool of normal pituitary. The lowest median expression of Cx32 was observed in NF-PitNETs, followed by somatotropinomas and corticotropinomas (Table 2).

Cx43 was the connexin that was most frequently expressed in PitNetETs adenomas. It was found in 58 of the 60 (97%) pituitary adenomas studied; 29 of the 31 (94%) NF-PitNETs, 20 of the 20 (100%) somatotropinomas, and 9 of the 9 (100%) corticotropinomas. The median Cx43 expression was lower in NF-PitNETs and somatotropinomas compared with the pool of normal pituitary. However, in corticotropinomas, an expression that was similar to the pool of normal pituitary was observed (Table 2). No statistically significant differences were found among the median expression of Cx26, 32, and 43 and PitNETs clinical features, such as age, gender, tumor type, tumor size, and invasiveness.

### 3.2. Protein Expression of Connexin 43 in Normal Human Pituitary and Pituitary Neuroendocrine Adenomas

**Figure 1:**
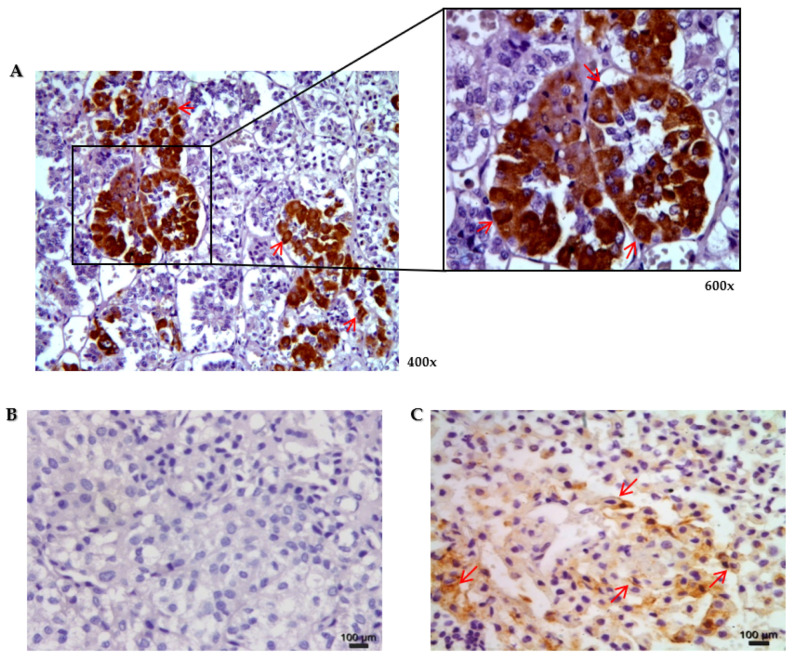
Representative microphotographs of immunohistochemical staining for connexin 43 (Cx43) in normal human pituitary and pituitary neuroendocrine tumors. (**A**)—The immunoreactivity of Cx43 was observed both in the cell membrane and/or in the cytoplasm in more than 50% of the cells of the adenohypophysis. (**B**,**C**)—Immunohistochemical staining for Cx43 in pituitary neuroendocrine adenomas. Variable immunoreactivity of Cx43 was observed in the cell membranes and/or in the cytoplasm of the pituitary adenomas. The red arrows highlight the presence of Cx43 membrane expression.

The protein expression of Cx43 in normal human pituitary and PitNETs was evaluated. We observed immunostaining for Cx43 in different samples of normal pituitary glands both in the cell membrane and/or in the cytoplasm. In adenohypophysis, immunopositivity was observed with strong intensity in more than 50% of the cells (Figure 1).

The protein expression was assessed by immunohistochemistry in 65 PitNETs (30 NF-PitNETs, 24 somatotropinomas, and 11 corticotropinomas). Immunoreactivity to Cx43 in PitNETs was classified as absent in 80% of the samples (no immunostaining or positive immunostaining in less than 5% of tumor cells).

In NF-PitNETs, Cx43 immunoreactivity was rarely observed, being absent in 26 (86%), low in 2 (7%), and moderate/high in 2 (7%) of the 30 cases. In somatotropinomas, Cx43 immunoreactivity was also rarely observed, being absent in 21 (87%) and low in 3 (13%) cases of the 24, none of the cases showing a moderate/high score. However, in the 11 corticotropinomas, Cx43 immunoreactivity was absent in 3 (27%), low in 3 (27%), and moderate/high in 5 (46%) cases. Therefore, Cx43 immunoreactivity was more frequently observed in corticotropinomas than in NF-PitNETs or somatotropinomas (Table 3). No association was found between Cx43 protein expression and clinical features, such as age, gender, tumor type, tumor size, and invasiveness.

### 3.3. Overexpression of Cx43 Decreases Cell Number in Pituitary Tumor Cell Lines

**Figure 2:**
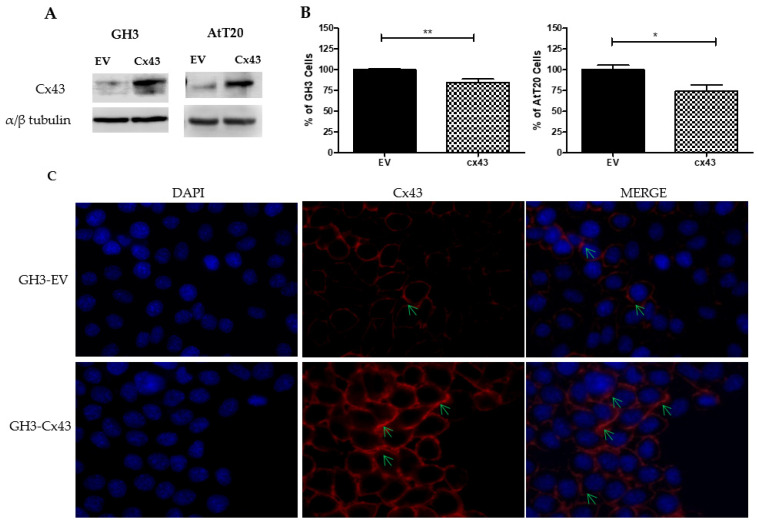
Effect of connexin 43 overexpression in GH3 and AtT-20 cell lines. (**A**)—representative Western blot of Cx43 in GH3 and AtT-20 cells that were transfected with Cx43 and empty vector (EV). (**B**)—Graphic representation of the number of cells in GH3-Cx43 and AtT-20-Cx43 compared with the controls GH3-EV and AtT-20-EV, respectively. The data are presented as the mean ± SEM and representative of at least three experiments that were performed in duplicate (calculated using Mann–Whitney U-test). * represents significant differences (0.01 ≤ *p* < 0.05), ** represents very significant differences (0.001 ≤ *p* < 0.01). (**C**)—Immunofluorescence of Cx43 (red) in GH3-EV and GH3-Cx43 cells, nuclear staining with DAPI (blue), at 400x magnification. The green arrows indicate the presence of Cx43 in gap junctions between cells.

In this work we used two rodent-derived cell lines, GH3, a secreting growth hormone and prolactin cell line, and AtT-20, a secreting adrenonorticotropic hormone cell line. The GH3 cells were transfected with the empty vector MClover2-N1 or with the vector mClover2-Cx43-7 containing the *Rattus norvegicus* connexin 43 coding sequence. The AtT-20 cells were transfected with the empty vector MClover2-N1 or with the vector mClover2-Cx43-7 modified by directed mutagenesis in part of the Cx43 sequence (see the Material and Methods section). The transfection allowed the evaluation of the effect of overexpressing Cx43 in the two cell lines. The transfection efficiency in both cell lines was verified by immunoblotting and by immunofluorescence using the anti-connexin 43 antibody (Figure 2). Transfection of CX43 resulted in the overexpression of Cx43 in both cell lines (Figure 2A).

In GH3 cells that were transfected with Cx43, GH3-Cx43, a significant reduction in the number of cells was observed when compared with GH3 cells that were transfected with the empty vector, GH-EV (*p* = 0.003) (Figure 2B). In GH3-Cx43 cells, the occurrence of gap junction plaque structures was evident, whereas GH3-EV cells showed weak Cx43 staining (Figure 2C). In AtT-20 cells that were transfected with Cx43, AtT-20-Cx43, a significant reduction in the number of cells was also observed when compared with AtT-20 cells that were transfected with the empty vector (*p* = 0.04) (Figure 2B).

### 3.4. Overexpression of Cx43 Affects the Cell Cycle in Pituitary Tumor Cell Lines

**Figure 3:**
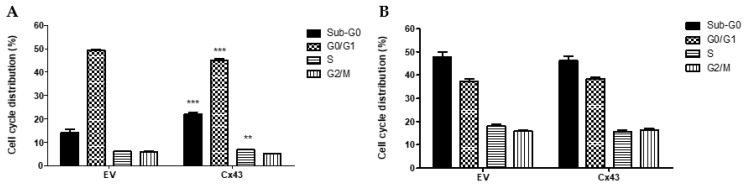
Effect of connexin 43 overexpression in the cell cycle of GH3 and AtT-20 cell lines that were transfected with empty vector (EV) or Cx43. (**A**)—Cell cycle distribution of transfected GH3 cells. (**B**)—Cell cycle distribution of transfected AtT-20 cells. The data are presented as the mean ± SEM and are representative of at least three experiments that were performed in triplicate, analysis using Mann–Whitney U-test. ** represents very significant differences (0.001 ≤ *p* < 0.01), *** represents extremely significant differences (0.0001 ≤ *p* < 0.001), comparing which cycle phase of Cx43 with control (EV).

To evaluate the effects of overexpression of Cx43 in cell cycle progression in pituitary cell lines, the cell cycle profile of GH3 and AtT-20 cells that were transfected with Cx43 or empty vector was analyzed. We observed that GH3-Cx43 cells had a significant increase of approximately 6% in the subG0 phase (*p* < 0.0001), a significant decrease of approximately 4% in the G0/G1 phase (*p* < 0.0001), and an increase of approximately 3% in the S phase (*p* = 0.007), compared to the respective phases of cell cycle of GH3-EV cells (Figure 3A).

In AtT-20-Cx43 cells, the distribution of the cell population among AtT-20 cells that were transfected with Cx43 or empty vector showed no statistically significant differences (Figure 3B).

### 3.5. Overexpression of Cx43 Increases the Number of Apoptotic Cells in Pituitary Tumor Cell Lines

**Figure 4:**
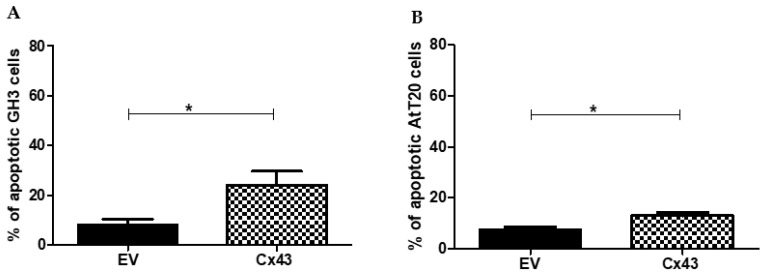
Effect of connexin 43 overexpression in the apoptosis of GH3 and AtT-20 cell lines. (**A**)—Graphic representation of the percentage of apoptotic cells in GH3-EV and GH3-Cx43. (**B**)—Graphic representation of the percentage of apoptotic cells in AtT-20-EV and AtT-20-Cx43. The data are presented as the mean ± SEM and are representative of at least three experiments that were performed in duplicate (analysis using Mann–Whitney U-tes.t, * *p* < 0.05).

The percentage of apoptotic cells in GH3 and AtT-20 cells that were transfected with Cx43 or EV was measured. We observed a significant increase in apoptosis of cells that were transfected with Cx43 compared with cells that were transfected with the empty vector in both GH3 and AtT-20 cells (Figure 4).

In the GH3 cells, the mean number of apoptotic cells was approximately 20% higher in GH3-Cx43 cells than in GH3-EV cells (*p* = 0.0063) (Figure 4A).

In the AtT-20 cells, the number of apoptotic cells was also significantly higher in AtT-20-Cx43 cells than in AtT-20-Cx43-EV cells (*p* = 0.0112) (Figure 4B).

## 4. Discussion

Pituitary neuroendocrine tumors (PitNETs) represent 15–20% of all diagnosed human brain tumors [24]. PitNETs are usually benign but can locally invade into adjacent tissues, such as the cavernous sinus and dura-mater. Some of these invasive tumors exhibit varying degrees of resistance to standard therapy and tend to recur. Early prediction of which pituitary neuroendocrine tumors will recur and/or exhibit an invasive phenotype remains difficult, despite the introduction of several tissue-based molecular markers [25,26,33] Therefore, understanding the role of other molecular markers in pituitary tumorigenesis can be important for predicting tumor behavior either before and/or after surgical resection and predicting the pharmacological response.

The present study evaluates the mRNA and protein expression of connexins in pituitary neuroendocrine adenomas. Meda et al. [34] reported the presence of Cx26 and 43, but not of Cx32, in the pituitary. Yamamoto et al. [35] demonstrated through immunohistochemical studies that Cx43 is the major connexin subtype that is expressed in the rat pituitary, being preferentially expressed in FS cells and gonadotrophs cells. In 1996, Morand et al. [23] demonstrated the presence of gap junctions between anterior pituitary cells of the same type and between different types of anterior pituitary cells, having distinct functions. Although numerous studies have suggested that gap junctions are an integral component of glandular cell-cell communication, the intercellular communication mechanisms in the anterior pituitary remain poorly characterized [10]. The presence of a functional junctional gap coupling between GH cells had been initially reported in guinea-pig pituitary slices by Guerineau et al. [36] and functional coupling between FS cells has been described in 2001 by Fauquier et al. [37].

The mechanism of the reduction in connexin expression and the consequent loss of gap junction communication has been reported to be associated with the process of tumorigenesis in different cell types [5,11,13,38]. However, the process is not completely understood and might be due to increased degradation of gap junction channels or the reduced translation and/or post-transcriptional modifications of the protein [11,18]. Our results concerning connexin mRNA expression suggests that the downregulation of protein expression can occur at the translational level.

In our work, we found variable amounts of mRNA expression levels of the Cx26, 32, and 43, as well as the presence of different connexins in the same tumor sample. The mRNA levels of connexins were in general downregulated in the pituitary neuroendocrine adenomas compared to the pool of normal pituitaries. Many studies have described that the relationship between the reduction in connexin expression and the tumorigenesis process is not only due to connexins channel-forming capability but also because connexins affect other cellular events independent of GJIC formation [38,39,40]. Immunoexpression of the Cx43 protein in NF-PitNETs and somatotropinomas was rarely observed. These data corroborate previous studies that also found an important reduction in the expression and/or function of Cx43 in many solid tumors, both benign and malignant [10,11,17,18,20,41].

Corticotropinomas, however, present frequent expression of Cx43, both at the mRNA and protein level, suggesting a possible distinct role of Cx43 in the tumorigenesis process of this cell type. Yamasaki et al. [1] described that connexins genes could exert a differential cell growth control effect, depending on the cell type in which they are expressed. Further studies in corticotropinomas are necessary to disclose Cx43 role in these adenomas.

Our data reveals that overexpression of Cx43 induced a significant reduction in the number of cells of pituitary tumor cell lines, GH3 and AtT-20, indicating that Cx43 might regulate pituitary cell growth. Our results are in line with previous studies that suggest that overexpression of Cx43 can reduce cell proliferation in different tumor cells and modify the tumor phenotype [14,42]. Ionta et al. [42] showed that exogenous Cx43 expression leads to a decrease in the growth of rat hepatocellular carcinoma cells and contributes to the reversion of the transformed phenotype, proposing that these effects were independent of GJIC formation capacity and probably associated with changes in the phosphorylation pattern and/or redistribution of the Cx43 protein in cellular compartments. The immunolabeling pattern for Cx43 varies according to the cell type, especially in tumor cells, and does not necessarily appear on the cell membrane forming typical junctional plaques [43,44].

In the present study, we found that the overexpression of Cx43 affected the cell cycle profile in GH3 cells, corroborating previous studies in other models [14,45]. Zhang et al. [46] suggested that Cx43 can act as a tumor-suppressor gene and that Cx43 suppresses the transition from G1 to the S phase of the cell cycle via an increase in p27 level. In GH3-Cx43 cells, we observed a significant reduction in the percentage of cells in the G0/G1 phase and a significant increase in the S phase, and we did not find alterations in the percentage of cells in the G2/M phase. However, Cx43 overexpression did not alter the adrenonorticotropic hormone-secreting AtT-20 cell cycle.

Connexins, or at least specific parts of these molecules, have been suggested to be able to act in the cell nucleus to directly affect gene transcription, increase the propagation of death signals, induce enhanced sensitivity to cell death, and inhibit proliferation via the regulation of specific kinases [21,40,46]. Our results are in agreement with previous studies that reported that Cx43 could directly or indirectly regulate growth and apoptosis [40,47], as we also observed an increase in apoptosis after overexpression of Cx43, more pronounced in GH3 cells than in AtT-20, together with a reduction of the number of cells. Cx43 has been demonstrated to be associated with a wide spectrum of apoptotic genes, including those coding for Bcl-xL, Bak, Bax, Bid, Diablo, caspase 6, and caspase 9 [40,48].

Recent studies have proposed Cx43 as a possible prognostic marker, which may predict clinical outcomes in chemotherapy-treated patients [11,49,50]. These data support the therapeutic potential of targeting Cx43 in different types of tumors due to the fundamental importance of this protein in regulating tissue homeostasis, proliferation, differentiation, survival, and apoptosis [47,51]. Tittarelli et al. [20] showed an important role of Cx43 in the control of growth, death, and metastasis in melanoma and suggested the use of components that selectively increase Cx43 expression in future treatment protocols. Additionally, Wang et al. [52] demonstrated that altered Cx43 expression modulates pituitary tumor transforming gene expression, which correlates with prolactinoma development and may be of therapeutic interest as a molecular target.

In summary, the present study represents a first effort for understanding the role of connexin in the tumorigenesis of pituitary neuroendocrine tumors. We show downregulation of connexins in these adenomas, and that Cx43 overexpression impairs growth and promotes apoptosis in pituitary cell lines. Further studies are necessary for a better understanding of the contribution of gap junction communication and the expression of connexin in the tumorigenesis of pituitary adenomas.

## Figures and Tables

**Table 1:** Clinical-pathological features of patients with pituitary neuroendocrine tumors (PitNETs).

Clinical-Pathological Features		PitNETs (n)	
	NF-PitNETs *	Somatotropinomas	Corticotropinomas
Number of cases	38	27	13
Age (years) at surgery (n, %)			
≤45	15 (39)	19 (70)	9 (70)
>45	23 (61)	8 (30)	4 (30)
Gender (n, %)			
Female	15 (39)	14 (50)	12 (92)
Male	23 (61)	13 (50)	1 (8)
Tumor size (n, %)	30	19	7
Microadenomas	0 (0)	2 (11)	3 (43)
Macroadenomas	15 (50)	12 (63)	4 (57)
Giant adenoma	15 (50)	5 (26)	0 (0)
Invasiveness (n, %)	28	19	6
Noninvasive	9 (32)	4 (21)	0 (0)
Invasive	19 (68)	15 (79)	6 (100)

* NF-PitNETs—Nonfunctioning Pituitary Endocrine.

**Table 2:** mRNA expression levels (2^−ΔΔCt^) of connexin 26, 32, and 43 in pituitary neuroendocrine tumors (PitNETs).

	N. of Cases	Connexin 26	Connexin 32	Connexin 43
NF-PitNET *	31			
Median (min-max)		0.24 (0–24.51)	0.15 (0–23.15)	0.19 (0–4.49)
Somatotropinomas	20			
Median (min-max)		1.27 (0–14.91)	0.59 (0–13.47)	0.38(0–3.06)
Corticotropinomas	9			
Median (min-max)		0.5 (0–14.62)	0.61 (0–11.17)	1.03 (4.63)
Total	60			

* NF-PitNETs—Nonfunctioning Pituitary Endocrine.

**Table 3:** Immunoreactivity score of the protein expression of connexin 43 in pituitary neuroendocrine tumor (PitNET).

		Connexin 43 Expression		
Tumor Type	N. of Cases	Negative	Low	Moderate/High
NF-PitNETs *	30	26	2	2
Somatotropinomas	24	21	3	0
Corticotropinomas	11	3	3	5
Total	65	50	8	7

* NF-PitNETs—Nonfunctioning Pituitary Endocrine.

## Data Availability

The datasets that were generated during and/or analyzed in the present study are accessible from the corresponding author on reasonable demand.
